# Safety issues related to the electronic cross-matching of blood in mainland China: A prospective cohort study involving cross-matching of 40,228 blood samples

**DOI:** 10.1097/MD.0000000000016703

**Published:** 2019-08-30

**Authors:** Yuan-Jie Wang, Jia-Rui Liu, Yong Liu

**Affiliations:** a Blood Transfusion Department, Suining Central Hospital, An Affiliated Hospital of Chongqing University; b Clinical Laboratory, Suining Central Hospital, An Affiliated Hospital of Chongqing University; c Intensive Care Unit, Suining Central Hospital, An Affiliated Hospital of Chongqing University, Suining, Sichuan, China.

**Keywords:** antibody screening, antigenic spectrum, electronic cross-matching, undetected antibody

## Abstract

Although the electronic cross-matching of blood has been widely applied in some developed countries and regions, concern over the risk of undetected red blood cell (RBC) antibodies has limited its application in mainland China.

This study was performed to explore the missed detection rate of RBC antibodies in a Chinese population from 2011 to 2016. If the results of 2 consecutive tests of ABO/RhD blood group identification were consistent and antibody screening results were negative, electronic cross-matching of the blood was performed. In addition, traditional serological cross-matching of blood (polybrene method) and a parallel experiment for electronic cross-matching of blood were performed to analyze the missed detection of unexpected RBC antibodies in blood donors and recipients.

Using the polybrene method, 40,228 blood samples were tested by parallel traditional serological cross-matching of blood; among these samples, blood compatibility was found in 40,222 cases, primary incompatibility (incompatibility of the donor's erythrocytes with the recipient's serum) was found in 6 cases, and no secondary incompatibility was found. Identification of antibody specificity was performed using panel cells, and all unexpected RBC antibodies were confirmed as anti-Mur alloantibodies in the MNS system.

Further improvements in the erythrocyte antigenic spectrum, especially the Mur antigen in Asian populations, are expected to ensure the safety of implementing electronic cross-matching in China.

## Abstract 2

Supplemental Digital Content is available in the text

## 1 Introduction

With the application and development of information technology in the field of blood transfusion medicine, automatic identification, and electronic blood-matching, and electronic blood-issuing systems have emerged. Electronic cross-matching is based on blood group identification and antibody screening for erythrocyte blood groups (referred to as antibody screening) to directly select blood with compatible ABO/RhD blood groups for each patient using a computer system, without serological cross-matching of blood for the recipient. This technology has been widely applied in some developed countries and regions and has played an important role in improving the safety of blood transfusion. Compared to traditional serological cross-matching, this technique has many advantages, including its rapid processing time, low cost, and simple operation.^[1]^ Kulkarni et al concluded that electronic cross-matching is more secure than the traditional anti-globulin method; information verification and blood issuing can be performed using a computer, not only reducing the blood transfusion costs but also significantly simplifying the manual operation. As reported by the University of Michigan Medical Center, no ABO-incompatible blood transfusions have been found in 138,000 cases of electronic cross-matching.^[2]^ Electronic cross-matching is a new technology in which ABO/RhD identification and antibody screening for donors and recipients in clinical applications are the key steps in safety management and the main measures to ensure compatibility between donor and recipient blood. In the case of matched blood groups for donor and recipient blood, a negative result in antibody screening is necessary to avoid alloimmune responses in blood recipients. Because differences exist in the expression of red blood cell (RBC) blood group antigens between Asians and Caucasians, using an imported erythrocyte reagent for antibody screening as the key to electronic cross-matching is not suitable for the Chinese population. In addition, mainland China has lagged behind the implementation of this technology, leading to a lack of technical standards for electronic cross-matching. Thus, we performed blood group detection and antibody screening for blood donors and recipients following the foreign technical specifications of electronic cross-matching in blood management. The missed detection rate of unexpected RBC antibodies was analyzed to investigate the role of antibody screening using electronic cross-matching technology and its significance in ensuring the safety of blood transfusion.

## 2 Methods

### 2.1 Main instruments

The automatic blood group identification and cross-matching blood analyzer and the blood group workstation were purchased from Johnson & Johnson, USA. The centrifuge used by the blood bank was obtained from BASO, Taiwan.

### 2.2 Detection reagents

Microcolumn agglutination cards (immunoglobulin G (IgG) and complement component 3d (C3d) test cards, blood type identification card) were obtained from Johnson & Johnson, USA. The erythrocyte reagents for antibody screening were obtained from 4 different manufacturers, including 2 imported erythrocyte reagents and 2 domestic erythrocyte reagents (Shanghai Blood Biomedicine Co. Ltd., China; Jiang Su Libio Medicine Biotechnology Co. Ltd., China; Ortho-Clinical Diagnostics Bermuda Co. Ltd., UK; DiaMed GmbH, Switzerland). Ten types of panel cells were used. The anti-A, anti-B, and anti-D reagents were purchased from Shanghai Hemo-Pharmaceutical & Biological Co., Ltd. The Baso-polymatching reagent kits for the polybrene method were obtained from BASO, Taiwan.

### 2.3 Blood transfusion management system

The intelligent software system for the quality management of safe blood transfusion was purchased from Chongqing Tupo Information Technology Co., Ltd.^[3]^ The module for the electronic cross-matching function was developed following foreign standards, and the automatic blood group identification and cross-matching blood analyzer was connected to the system.

### 2.4 Blood samples from blood donors

A red cell suspension from blood donors was used to prepare the blood samples from which blood clots were removed with a clean piece of bamboo. Then, the suspension liquid was centrifuged at 3000 r/min for 5 minutes to isolate the plasma and red cells for cross-matching.

### 2.5 Blood samples from blood recipients

This prospective observational study was conducted from 2011 to 2016, and all participants were Han Chinese. The experimental protocol was approved by the Ethics Committee of Suining Central Hospital. Informed consent was waived due to the nature of observational studies. For the blood samples from blood recipients, 2.0 to 3.0 ml was collected from each inpatient and outpatient of our hospital who underwent blood transfusions and received blood during surgery. Plasma specimens were isolated via centrifugation at 3000 r/min for 5 minutes after ethylenediaminetetraacetic acid (EDTA) anticoagulation. Blood samples from blood recipients were collected within 3 days of a transfusion; blood samples were recollected if retransfusion was performed more than 3 days after cross-matching. The blood samples were stored in a refrigerator at 2 to 6 °C before use.

### 2.6 Detection of the ABO and RhD blood group in blood donors and blood recipients

The ABO and RhD blood group positivity and negativity of blood donors were first identified using the saline medium method to determine the accuracy of the blood group.^[4]^ Antibody screening was performed with an automatic blood group identification and blood cross-matching analyzer. Samples with negative results were automatically put into the intelligent software system for quality management of safe blood transfusions. Samples with positive results were returned to the blood bank. The ABO and RhD blood group positivity and negativity of blood recipients (except newborns) were identified using an automatic blood type identification and blood cross-matching analyzer. Because newborns do not produce the ABO blood group antibody or do so incompletely, the ABO blood group typing does not match in forward and reverse typing. Therefore, electronic blood matching is unsuitable for newborns. The results were automatically input into the intelligent software system for quality management of safe blood transfusion to establish blood group files for the patients. For patients who required blood transfusions or surgery, the blood samples were recollected for secondary blood group identification, including ABO and RhD blood group positivity and negativity, and routine antibody screening was performed by adopting 1 domestic erythrocyte reagent. Samples with negative results were automatically input into the intelligent software system for quality management of safe blood transfusion. Samples with positive results were input into the intelligent software system for quality management of safe blood transfusion after labeling, without implementing the electronic cross-matching.

### 2.7 Rules for electronic cross-matching

(1) Patients must have at least 2 matched results in the ABO/RhD blood group identification (electronic cross-matching should not be performed for cases with unmatched positive and negative ABO blood groups), and 1 of matched results must have been obtained from the current sample.
(2) Antibody screening of the patients must be negative, with no positive results in previous antibody screenings.
(3) The computer system must be able to prevent the release of incompatible blood.
(4) The computer system and other key equipment must be strictly checked and confirmed.
(5) Control procedures must be used to ensure accurate data input and automatic data transmission.
(6) The computer should be able to issue a warning for cases with historical information that does not conform to the electronic cross-matching conditions.
(7) Identification of the ABO/RhD blood group for the erythrocyte component of the donor's blood should be correct, with a negative result in antibody screening.^[3]^

### 2.8 Serological cross-matching

**Figure 1:**
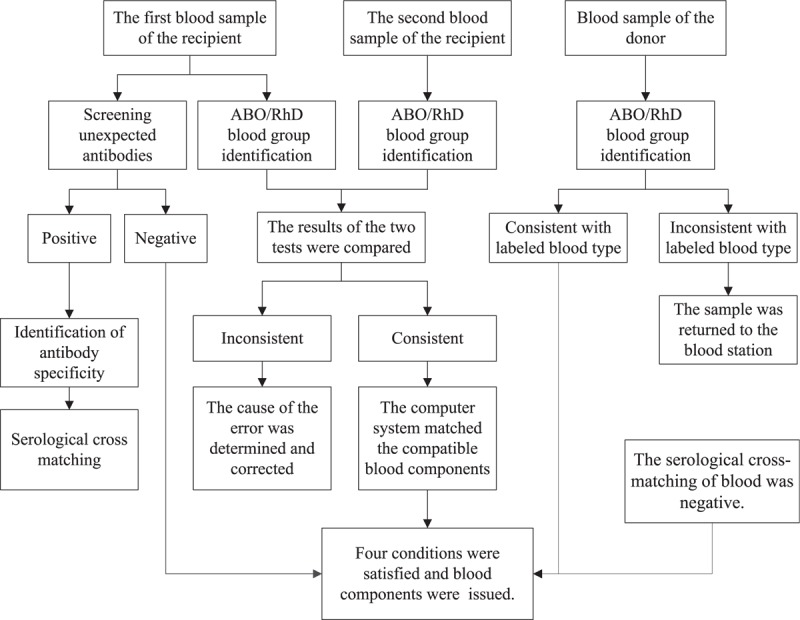
Flow chart for performing blood cross-matching.

For samples consistent with the electronic cross-matching rules, the intelligent software system for quality management of safe blood transfusion can automatically display the available donors according to the required blood composition and quantity, along with the inventory of blood, to complete the electronic cross-matching. Additionally, parallel testing by traditional serological cross-matching, using the polybrene method, was performed for blood samples from donors and recipients who met the criteria for electronic cross-matching. For samples showing incompatibility in the serological cross-matching test, another domestic and 2 imported erythrocyte reagents were used to screen the antibody again, and the results of the 3 reagents were recorded. Then, identification of antibody specificity was performed with panel cells to select compatible blood for the transfusion. The experimental process is shown in Figure 1.

### 2.9 Statistical analyses

Count data are expressed as rates (%). The Pearson Chi-squared test or the Fisher exact test were used to compare the rates of events between 2 groups. A *P* value less than .05 was considered statistically significant. All statistical analyses were completed by SPSS 18.0 software (IBM, Armonk, NY).

## 3 Results

### 3.1 Detection of blood groups in blood donors

The compliance rate of ABO/RhD blood group testing for donors between the blood station and our hospital was 100%. No cases with inconsistent results of 2 ABO/RhD blood group tests were found.

### 3.2 Antibody screening

Among the 40,630 blood samples from patients, antibody screening was positive in 247 (0.61%) cases, with various immune statuses, including a history of transfusion, pregnancy, or both, as shown in Table 1. Among the 27,535 blood samples from blood donors, antibody screening was positive in 19 cases, yielding a positive rate of 0.07%. The distribution of antibody specificity in the 247 antibodies screening-positive samples is shown in Table 2.

**Table 1:**
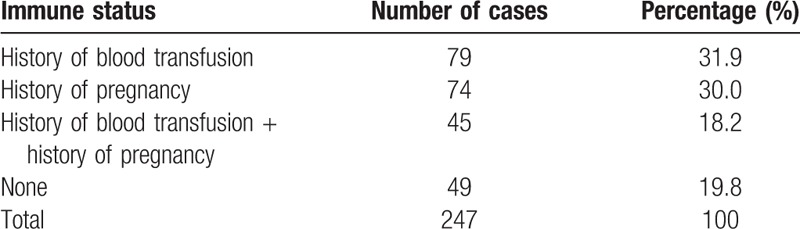
Statistics of 247 samples from patients with positive results in antibody screening.

**Table 2:**
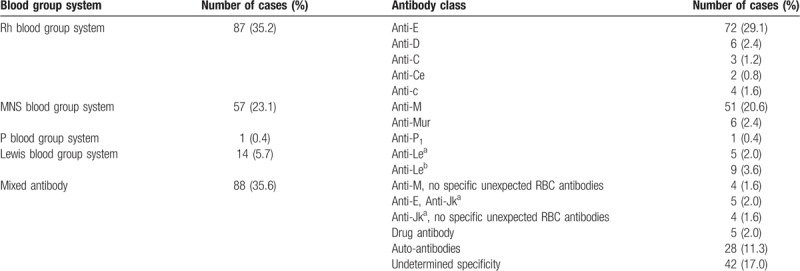
Antibody distribution of 247 samples from patients with positive results in antibody screening.

### 3.3 Cross-matching

Excluding 155 blood samples from newborns, 247 samples showed positive results in the antibody screening. The remaining 40,228 blood samples were consistent with the rules of electronic cross-matching, and no ABO/RhD incompatibilities were found in the electronic cross-matching implemented by the computer. Using the polybrene method, the blood samples were tested by parallel traditional serological cross-matching; among these samples, blood compatibility was found in 40,222 cases, primary incompatibility (incompatibility of the donor's erythrocytes with the recipient's serum) was found in 6 cases, and no secondary incompatibility (incompatibility of the recipient's erythrocytes with the donor's serum) was found. RBC components from 27,535 donors were used, with no occurrence of alloimmune responses. Further testing showed that the 6 incompatible blood samples contained unexpected RBC antibodies, resulting in a missed detection rate of 2.37% [6/(247 + 6)].

### 3.4 Identification of antibody specificity

The results of traditional serological cross-matching of blood by the polybrene method showed that 6 blood samples were incompatible with the blood donors. Identification of antibody specificity was performed using panel cells, and all unexpected RBC antibodies were confirmed as anti-manganese uptake regulator (Mur) alloantibodies in the MNS system. Then, Mur antibodies were used to identify Mur antigen-negative erythrocytes, which were provided for recipients with positive serological reactions.

## 4 Discussion

Blood compatibility testing before blood transfusion is performed to prevent an alloimmune response caused by an incompatible blood transfusion. Electronic cross-matching is different from traditional serological cross-matching as during electronic cross-matching, ABO/RhD blood group identification and antibody screening are performed for blood donors and recipients. The compatibility tests between donors and recipients are completed by a computer system under the premise of a matched blood group and negative antibody screening, without serological cross-matching. Using this method, secondary blood group testing is performed to ensure donor and recipient ABO/RhD compatibility, and antibody screening has become a key step in electronic cross-matching technology.^[5]^ However, antibody screening cannot detect all unexpected RBC antibodies, especially low-frequency antibodies, and alloimmune responses may occur in recipients. The probability of an alloimmune response is closely related to the antigen coverage and combination of antibody screening cells. Only when the missed detection rate of unexpected RBC antibodies is reduced to a minimal level can the safety and reliability of electronic cross-matching technology be ensured.

The "Guidelines for Compatibility Procedures in Blood Transfusion Laboratories” by the British Committee for Standardization in Haematology (BCSH) proposed detailed requirements for coverage of the antigen and antigen combinations in antibody screening cells based on the data of severe adverse reactions related to blood transfusion. Furthermore, if detection is performed strictly following these guidelines, the sensitivity of antibody screening is believed to be higher than that of the serological cross-matching test.^[6]^ Due to the improvement of laboratory management levels, the interlaboratory quality assessment by the UK National External Quality Assessment Service (NEQAS) showed that the missed detection rate in antibody screening decreased from 6.65% in 1984–1985 to 0.07% in 1996–1997.^[7]^ In 1990, Cordle et al reported comparison results of antibody screening and cross-matching tests in 3380 patients, with an approximately 0.6% rate of positive cross-matched blood in cases with negative antibody screening results.^[8]^ According to the estimation by Garratty et al, the missed detection rate of potentially clinically significant antibodies was approximately 1:17,000.^[9]^ According to studies in other countries, including Denmark, Italy, New Zealand, and France, the implementation of electronic cross-matching technology is safe and reliable.^[10]^ Although some hospitals in China have begun to use electronic cross-matching on the basis of serological cross-matching, it is difficult to implement widely because of the lack of corresponding reagents and guidelines.^[11,12]^

Due to the differences in erythrocyte blood group antigens in different races, the coverage of antibody screening cells and the combination of cells are particularly important for the safety of blood transfusions in the application of electronic cross-matching technology. For example, the expression of the Diego blood group is mostly limited to Mongolian and indigenous American populations,^[13]^ and Mur antigens are rare in Caucasians and Africans, while 7% of Chinese and 10% of Thai people are positive for these antigens.^[14]^ The antibodies corresponding to the antigens of these blood group systems can cause adverse reactions to hemolytic transfusion or invalid RBC infusion. In addition, some antibodies, such as anti-K, can cause severe hemolytic transfusion reactions and neonatal hemolytic disease, while anti-Le^b^ can cause damage to transfused Le(b+) erythrocytes, resulting in hemolytic transfusion reactions.^[15]^

MNS hybrid glycophorins, comprising a series of low-frequency antigens, derive from allele rearrangement between glycophorin A (GYPA), glycophorin B (GYPB), and sometimes glycophorin E (GYPE). The Mur antigen, the GYP (B-A-B) hybrid gene, is generated by the homologous sequence of GYPA replacing part of the GYPB sequence. The 4 Mur-positive hybrid glycophorins include GP. Mur (Mi. III), GP. Bun (Mi. VI), GP. Hop (Mi. IV) and GP. Kip; GP. Mur (Mi. III) is the most common hybrid glycophorin in Southeast Asia.^[16]^ In the present study, the erythrocyte reagents used for antibody screening were products commonly used in clinical practice from 4 manufacturers, including 2 imported and 2 domestic erythrocyte reagents. The positive rate of antibody screening was 0.61%, similar to the findings reported by Chi et al.^[17]^ The 6 cases of missed detection of unexpected RBC antibodies were determined to be anti-Mur antibodies. However, using the antibody screening cells from the 4 manufacturers resulted in a missed detection rate of 2.37%, which is much higher than that reported in the literature.^[7]^ A domestic erythrocyte reagent was able to detect 8 blood group systems, but "uncertainty” was found for the Mur, Di^a^, and Di^b^ antigens. Another domestic erythrocyte reagent could detect 7 blood group systems, but the Mur antigen was not labeled in the manufacturer's specifications. Although the 2 imported erythrocyte reagents could detect 9 blood group systems, the Diego blood type system and the Mur antigen for detecting Mongolian samples were not included in the 2 products. For successful electronic cross-matching in Mongolians, antibody screening cell antigens should cover the Mur, Di^a^, Di^b^, Le^b^, and K antigens due to their high frequency to minimize the missed detection rates of these unexpected RBC antibodies, thereby ensuring the safety of blood transfusion among these patients.

As electronic cross-matching technology has not yet been implemented in mainland China, relevant technology is lacking, and no national standards for the coverage of antigens or the combination of antibody screening cells are available. Both domestic and imported erythrocyte reagents for clinical antibody screening in mainland China have the risk of high missed detection rates of unexpected RBC antibodies and cannot ensure safety in the application of electronic cross-matching technology. At present, automation, informatization and laboratory quality management have been improved in many large-scale hospitals in mainland China, and the basic conditions are in place for the implementation of electronic cross-matching. The development of standards for erythrocyte reagents in antibody screening is necessary as soon as possible for the clinical development of electronic cross-matching technology. As a relatively new blood transfusion technique, electronic cross-matching can shorten matching time and reduce the cost of reagents and the number of employees. In addition, this technique can reduce the number of experimental tests, provide reports in a timely manner, and reduce work records to prevent the incorrect issuing of blood components. A survey by Engelfreit et al showed that many countries and institutions have implemented or have planned to implement electronic cross-matching technology.^[18]^ Further improvements in the erythrocyte antigenic spectrum, especially the Mur antigen in Asian populations, are expected to ensure the safety of the implementation of electronic cross-matching in China.

Additional information on this study is provided on the website of Medicine.

## Acknowledgments

We owe special thanks to Dr. Cao and Dr. Gong for their kind help with the study design and manuscript writing.

## Author contributions

**Conceptualization:** Yuan-Jie Wang.

**Data curation:** Yuan-Jie Wang.

**Formal analysis:** Yuan-Jie Wang, Jia-Rui Liu.

**Funding acquisition:** Yuan-Jie Wang.

**Investigation:** Yuan-Jie Wang.

**Methodology:** Yuan-Jie Wang, Jia-Rui Liu.

**Project administration:** Jia-Rui Liu.

**Resources:** Jia-Rui Liu, Yong Liu.

**Software:** Yong Liu.

**Supervision:** Yong Liu.

**Validation:** Yong Liu.

**Visualization:** Yong Liu.

**Writing – original draft:** Yuan-Jie Wang, Yong Liu.

**Writing – review & editing:** Jia-Rui Liu, Yong Liu.

## Supplementary Material

Supplemental Digital Content
